# Using Light Quality for Growth Control of Cucumber Seedlings in Closed-Type Plant Production System

**DOI:** 10.3390/plants9050639

**Published:** 2020-05-17

**Authors:** Hyeon Woo Jeong, Hye Ri Lee, Hyeon Min Kim, Hye Min Kim, Hee Sung Hwang, Seung Jae Hwang

**Affiliations:** 1 Division of Applied Life Science, Graduate School of Gyeongsang National University, Jinju 52828, Korea; j_dk94@naver.com (H.W.J.); dgpfl77@naver.com (H.R.L.); 2 Division of Plant Resources, Korea National Arboretum, Yangpyeong 12519, Korea; hmk0766@korea.kr; 3 Protected Horticulture Research Institute, National Institute of Horticultural and Herbal Science, Rural Development Administration, Haman 52054, Korea; hmk5525@korea.kr; 4 Division of Crop Science, Graduate School of Gyeongsang National University, Jinju 52828, Korea; uldangc@naver.com; 5 Department of Agricultural Plant Science, College of Agriculture & Life Sciences, Gyeongsang National University, Jinju 52828, Korea; 6 Institute of Agriculture & Life Science, Gyeongsang National University, Jinju 52828, Korea; 7 Research Institute of Life Science, Gyeongsang National University, Jinju 52828, Korea

**Keywords:** chemical plant growth regulator, compactness, Fr light, hypocotyl length, UV-A

## Abstract

During seedling production, growth control of seedlings is an important problem because the overgrowth of seedlings causes a decrease of seedling quality and has disadvantages after transplanting. In this study, we aim to evaluate the possibility of replacing chemical plant growth regulators using light quality in a closed-type plant production system (CPPS) for cucumber seedling production. We used various light treatments, such as monochromatic or combined red (R) and blue (B), and combined R and B with UV-A or Far-red (Fr) light, to compare with a chemical plant growth regulator conventionally using in nursery farms. The combined R and B treatment decreased stem elongation and increased dry matter and compactness. UV-A treatment increased compactness but did not significantly affect the stem elongation or dry matter. Fr increased stem elongation and stem diameter and decreased compactness and dry matter. In leaf growth, combined R and B treatments and UV-A treatments increased leaf area, specific leaf weight, and SPAD value, and decreased leaf shape index. Fr treatments increased leaf area and leaf shape index and decreased specific leaf weight (SLW) and SPAD values. Cucumber seedlings have many different morphological changes, and R5B5 light quality was more effective in growth control due to higher compactness than chemical plant growth regulators. Also, R5B5 light quality has increased seedling quality, such as dry matter and SLW compared with fluorescent lamps. Thus, the use of light quality is a possible alternative to a chemical plant growth regulator.

## 1. Introduction

Crop growth controls are an important aspect during the culture period; controlling the growth of seedling requires delicate management. The excessive elongation of seedlings creates many problems, such as raising the position of the flower cluster, overgrowth after transplanting, poor fruiting, and fruit abscission during the fruiting season. This excessive elongation generally occurs with the low light intensity and short photoperiod in winter seasons and rainy periods in the summer season. Traditionally, to solve this problem, many commercial nursery farms use plant growth regulators. However, given regulatory restrictions on plant growth regulators, the increasing cost of chemicals, and the possibility of environmental pollution, alternative growth control methods are needed [1].

Many different alternatives, such as mechanical stimulation, temperature control, and irrigation control, are available to replace chemical plant growth regulators [2,3]. Mechanical stimulation is the favored method for growth control and automation [4]. However, excessive mechanical stimulation may lead to plant wounding, and this method is not appropriate for horticultural crops. Using differences between day and night temperatures (DIF) is another method for plant growth control [5]. However, this method cannot be realistically applied to nursery farms, which mainly use unenclosed greenhouses. Deficit irrigation makes plants smaller [6], but this method risks plant death or decreased plant quality. Therefore, research on safe and easy methods for growth control is required.

Light is an important factor affecting plant growth and morphogenesis. In recent years, advances in LED technology have been used to apply various light spectra to agriculture. Different light quality induces different responses in plants. A previous study showed that different ratios of combined red (R) and blue (B) lights have different effects on plants [7,8,9,10,11]. Hernández and Kubota [7] tested different red and blue ratios on the growth and physiological characteristics of cucumber. They found that plant height, hypocotyl length, net photosynthetic rate (Pn), and stomatal conductance (g_s_) increased with B light in combined R and B light. Nanya et al. [8] reported that tomato dry mass is affected by different R and B light ratios. In many cases, different ratios of combined R and B light control plant elongation and leaf growth [9,10,11]. Therefore, light quality can be effectively used to control plant growth.

Ultraviolet (UV) light and far-red (Fr) light, a kind of invisible light, affect plant morphology. Generally, UV light is known to make the plant compact and stockier [12]. The ratio of R and Fr light can be used to control plant elongation [13]. However, many studies about UV and Fr light effects on plant morphology mostly used supplemental lighting with other light quality. Information is limited about combined R and B light with UV and Fr light.

Cucumber, an important horticultural crop, is sensitive to light quality treatments. Savvides et al. [14] found that cucumber had lower stomatal conductance (g_s_), hydraulic conductance, and net photosynthesis rate (Pn) when cultivated with monochromatic red light. Hogewoning et al. [15] tested various blue and red light ratios and found that when blue light was increased to 50R:50B, the leaf photosynthetic capacity, Pn, g_s_, and chlorophyll concentration increased. Also, there are many studies of UV and Fr light effects on cucumber plants [16,17,18,19]. However, in many cases, studies of these light qualities often focused on plant response in greenhouse conditions or supplemental use with sunlight, not in controlled conditions. Indeed, there is very little information on cucumber seedlings under combined R and B light with UV and Fr light.

Therefore, the aim of this study is to investigate the effect of combined R and B light with UV and Fr light and evaluate the possibility of applying various light spectra using LED as a replacement for chemical plant growth regulators in a CPPS.

## 2. Materials and Method

### 2.1. Plant Materials and Growth Conditions

Seeds of cucumber (*Cucumis sativus* L. “Joeunbaekdadagi”) were sown in 40-cell plug trays (54 × 28 × 4.8 cm, Bumnong Co. Ltd., Jeongeup, Korea) filled with a commercial growing medium on 28 June 2018, and grown in a closed-type plant production system (CPPS, C1200H3, FC Poibe Co. Ltd., Seoul, Korea) at 27 °C, 50% relative humidity (RH), and maintained in darkness during the germination period (4 days). After germination, 3 replicates of 20 seedlings per treatment were used for this experiment. The growth conditions were set to 25 ± 1 °C, 50% ± 10% relative humidity (RH), and 200 µmol·m^−2^·s^−1^ photosynthetic photon flux density (PPFD) on the basis of the trays’ height with a 12/12 h (light/dark) photoperiod. The light intensity was measured using a photometer (HD2101.2, Delta Ohm SrL, Caselle, Italy). The nutrient solution (Table 1) was supplied by sub-irrigation every second day, at 6.5 pH and 1.5 dS·m^−1^ electrical conductivity (EC). Plug trays were rearranged every day after irrigation to provide uniformly distributed light wavelengths and intensities. Seedlings were grown for 22 days after treatment (DAT) under various light treatments.

### 2.2. Light Qualtiy Treatments

**Figure 1:**
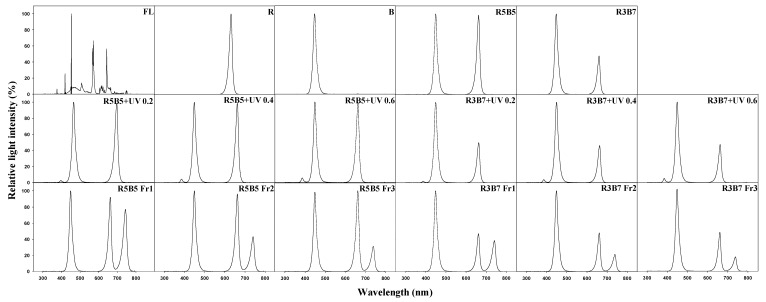
The spectral distribution of light used in the experiments.

To examine how combined red and blue light, UV-A, and far-red (Fr) light affect the growth of cucumber seedlings, fluorescent lamp (FL) and FL with foliar spray of diniconazole (Dini) (Binnali, Dongbang Agro Co. Ltd., Seoul, Republic of Korea) were used as the control. To compare the height suppression effects of chemical plant growth regulator, seedlings in Dini were supplied diniconazole as a single 150 mg·L^−1^ foliar spray when first true leaves appeared on a seedling. The different light treatments for this experiments were as follows: red:blue = 100:0 (R), red:blue = 50:50 (R5B5), red:blue = 30:70 (R3B7), or red:blue = 0:100 (B), UV-A light added to R5B5 and R3B7 (R5B5 + UV-A 0.2 W·m^−1^ (R5B5 UV 0.2), R5B5 + UV-A 0.4 W·m^−1^ (R5B5 UV 0.4), R5B5 + UV-A 0.6 W·m^−1^ (R5B5 UV 0.6), R3B7 + UV-A 0.2 W·m^−1^ (R3B7 UV 0.2), R3B7 + UV-A 0.4 W·m^−1^ (R3B7 UV 0.4), and R3B7 + UV-A 0.6 W·m^−1^ (R3B7 UV 0.6)), and far-red light added to R5B5 and R3B7 (R:B:Fr = 33:33:33 (R5B5 Fr1), R:B:Fr = 40:40:20 (R5B5 Fr2), R:B:Fr = 43:43:14 (R5B5 Fr3), R:B:Fr = 20:60:20 (R3B7 Fr1), R:B:Fr = 27:60:13 (R3B7 Fr2), and R:B:Fr = 30:60:10 (R3B7 Fr3)). The PPFD of each light treatment was determined from bandwidth integration (Table 2). The light spectral distribution was measured using a spectroradiometer (ILT950, International Light Technologies Inc., Peabody, MA, USA) at five points (center and four edges) on the top of the tray (Figure 1).

### 2.3. Measurements of Growth Characteristics

Plant growth parameters, such as the plant height, hypocotyl length, stem diameter, leaf area, leaf shape index, specific leaf weight (SLW), SPAD value, fresh and dry weights of stems and leaves, dry matter, and compactness were measured on 22 DAT. The stem diameter was measured 1 cm above the medium surface using digital Vernier calipers (CD-20CPX, Mitutoya Corp., Kawasaki, Japan). The leaf area was measured using a leaf area meter (LI-3000, LI-COR Inc., Lincoln, NE, USA). The SPAD value was measured on the second leaf from the top using a chlorophyll meter (SPAD-502, Konica Minolta Inc., Osaka, Japan), immediately prior to harvesting the samples. The fresh weight was measured with an electronic scale (EW 220-3NM, Kern & Sohn GmbH., Balingen, Germany). The dry weight was measured after drying the divided samples of the stems and leaves for 72 h in a drying oven (Venticell-222, MMM Medcenter Einrichtungen GmbH., Munich, Germany) at 70 °C.

The leaf shape index, SLW, dry matter, and compactness were calculated using the following equations:

Leaf shape index = leaf length/leaf width

SLW (mg·cm^−2^) = dry weight of leaf (mg)/total leaf area (cm^2^)

Dry matter (%) = (dry weight of shoot (g)/fresh weight of shoot (g)) × 100

Compactness (mg·cm^−1^) = dry weight of shoot (mg)/plant height (cm)

### 2.4. Statistical Analysis

The experiment was repeated three times, with twenty plants each in a completely randomized block design, and fifteen plants per treatment were used to determine plant growth parameters. The statistical analysis was carried out using the statistical analysis system program (SAS 9.1, SAS Institute Inc., Cary, NC, USA). The experimental results were subjected to an analysis of variance (ANOVA) and Duncan’s multiple range tests. Graphing was performed with the SigmaPlot program (SigmaPlot 12.0, Systat Software Inc., San Jose, CA, USA).

## 3. Results and Discussion

**Figure 2:**
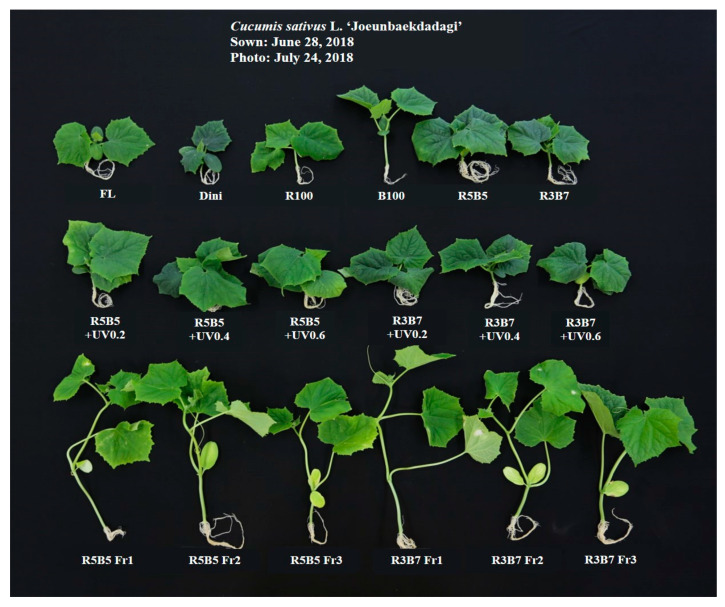
“Joeunbaekdadagi” cucumber (*Cucumis sativus* L.) plug seedlings grown under various light qualities at 22 days after treatment.

**Figure 3:**
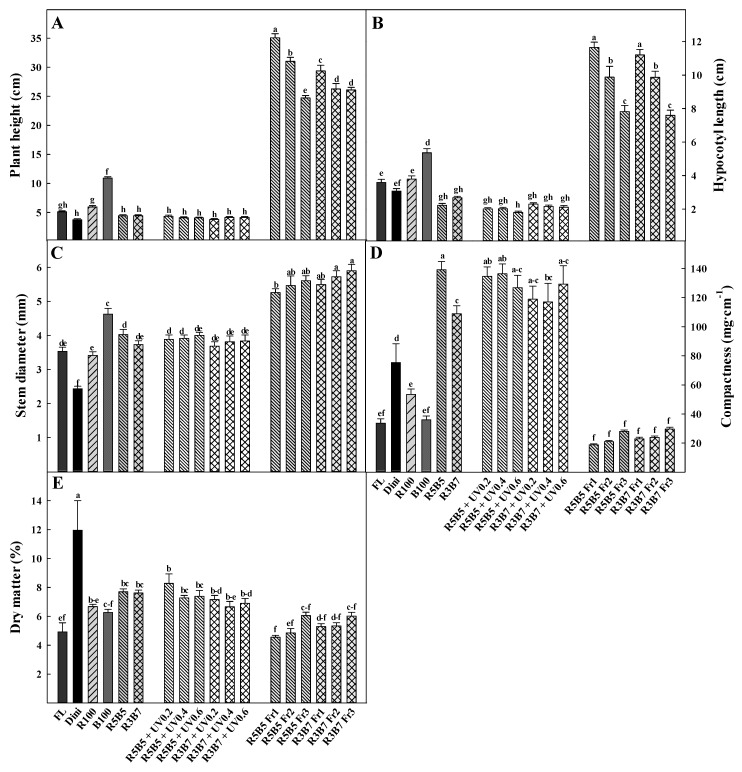
The effects of the light quality on (**A**) plant height, (**B**) hypocotyl length, (**C**) stem diameter, (**D**) compactness, and (**E**) dry matter of “Joeunbaekdadagi” cucumber (*Cucumis sativus* L.) plants measured at 22 days after treatment. Vertical bars indicate the mean ± SD. (*n* = 15). Different letters above the bars indicate significant differences by Duncan’s multiple range test at *p* ≤ 0.05.

The plant height and hypocotyl length of the cucumber seedlings are presented in Figure 2 and Figure 3, respectively. Plants grown under far-red light were taller and had longer hypocotyls than in non-Fr light treatment. The R5B5 Fr1 treatment had the tallest plant height and the longest hypocotyl length (Figure 3A,B). Except for the Fr light treatment, plants under blue light had the longest hypocotyls, and combined R:B and UV-A treatments had the shortest plant height and hypocotyls. Hernández and Kubota [7] reported that plant height and hypocotyl length of cucumber seedling under B light were greater than combined R and B light because monochromatic B light has a lower phytochrome photostationary state. UV-A intensity did not affect plant height or hypocotyl length. The plants under the Fr light treatment showed that both decreased plant height and hypocotyl length with increasing of R/Fr ratio. An earlier study reported that a low R/Fr ratio induces the shade avoidance response in plants, with the result that the plants have morphological changes, such as stem elongation, thinner leaves, and production of less dry matter [20,21]. In the present study, stem elongation of cucumber seedling under Fr light is considered the result of the shade avoidance response caused by Fr light. The stem diameter of cucumber seedlings had a different tendency with plant height and hypocotyl (Figure 3C), increasing with the increase in R/Fr ratios, which were the thinnest in Dini. Except for Fr treatments, the seedlings under blue light treatment had the thickest stem diameters. In UV-A treatments, the stem diameter was not significantly different by UV-A intensity. In our previous study, stem diameter of tomato seedling was increased under Fr light treatment; however, there are non-significant differences in R/Fr ratios [22]. It seems that there are species differences in tomato and cucumber plants.

The compactness of cucumber seedlings was the opposite of plant height and stem diameter (Figure 3D). R5B5 treatment had the greatest and Fr treatment had the lowest compactness. Blue light treatment was less effective than red light. The UV-A treatment produced greater compactness than the other treatments, such as FL, Dini, R, B, and Fr. Seedling compactness is the ratio of the shoot dry weight and the plant height, and high compactness means the seedling was short and stockier. In the present study, R5B5 treatment has more effective than Dini treatment on compactness; it means the R5B5 treatment has advanced for overgrowth retardant of cucumber seedling.

Dry matter was the highest in Dini, and compared with FL, combined R and B treatments had more of an effect on the dry matter than monochromatic R and B treatments (Figure 3E). In UV-A treatments, except for R5B5 + UV0.2, the dry matter tended to decrease in UV-A added treatments. Dry matter in Fr treatments increased with increasing of R/Fr ratio. In the previous study, the dry matter of tomato seedling also decreased under combined R and B with UV-A and Fr light [22]. Therefore, it is considered that the non-visible light reduces on dry matter of the seedlings. Dry matter is one of the important seedling quality factor [23]. Although the Dini treatment has the greatest Dry matter, combined R and B treatment and UV treatment has greater dry matter than FL. This result suggests that the light quality can increase the seedling quality of cucumber seedling. Brazaitytė et al. [24] tested cucumber seedlings under combined R and B and UV-A light, reporting that UV-A decreased the dry weight of cucumber seedlings. In the present study, compared with R5B5, the cucumber seedlings in R5B5 added UV-A treatments had lower dry weight (Table 3). The dry weight was the lowest in FL and the highest in Fr treatments. The dry weights of cucumber seedlings under Fr treatment increased with increasing of R/Fr ratio. Stutte [25] reported that leaf lettuce under far-red LEDs in combination with red light increased total biomass. To summarize, combined R and B treatments decreased stem elongation and increased dry matter and compactness. UV-A treatment increased compactness but did not significantly affect stem elongation or dry matter. Fr increased stem elongation and stem diameter and decreased compactness and dry matter.

**Figure 4:**
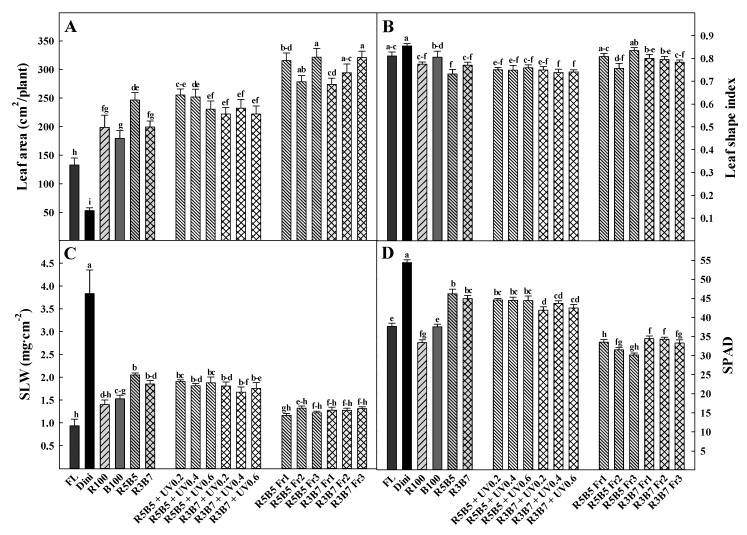
The effects of the light quality on (**A**) leaf area, (**B**) leaf shape index, (**C**) SLW, and (**D**) SPAD of “Joeunbaekdadagi” cucumber (*Cucumis sativus* L.) plants measured at 22 days after treatment. Vertical bars indicate the mean ± SD (*n* = 15). Different letters above the bars indicate significant differences by Duncan’s multiple range test at *p* ≤ 0.05.

Leaf area was the largest in Fr treatments and the smallest in the Dini treatment (Figure 4A). In UV-A treatments, the leaf area decreased with increasing UV-A intensity. Hernández and Kubota [7] reported that the leaf area of cucumber seedlings decreases with an increasing B ratio in combined R and B light. Our results also showed that the leaf area decreased with the increase of B ratio. Leaf area under R5B5 treatment was 24% larger than in R3B7. Early studies found that UV-A increases the total leaf area in *Glycine max* and some *Sorghum bicolor* [26,27]. However, in our results, only R3B7-added UV-A treatment increased leaf area, and R5B5 and R5B5 added UV-A treatments were not significantly different. Several studies reported that supplemental Fr light induces increases in the leaf area of lettuce [28,29,30]. Our results are the same as in previous studies: plants grown under Fr light have a larger leaf area.

The form of leaves is significantly related to the light environment. The leaf shape index was the largest in Dini and the lowest in R5B5 (Figure 4B). A higher leaf shape index indicates a longer leaf shape, and a lower leaf shape index indicates a squatter leaf. Another study reported that far-red light indicates leaf elongation [27]. In our results, the Fr treatment tended to have a higher leaf shape index value than combined R and B treatments. The leaf shape index of UV-A treatments was not significantly different between the different UV-A light intensity.

SLW is related to leaf thickness, and also one of the seedling quality factor [23]. Moreover, when the seedling has thicker leaves, it can hold more water in leaf [31], and it will be easily overcome drought stress occurs after transplanting. In this study, the SLW was the greatest in Dini and the lowest in FL (Figure 4C). The largest SLW in the Dini treatment seemed to be the result of the small leaf area. Combined R and B treatments and UV-A treatments had thicker leaves than FL, R, B, and Fr treatments. An earlier study reported that the SLW of *Phyllanthus tenellus* decreased with increasing UV-A intensity [29]. Also, Kim and Hwang [22] reported that SLW of tomato seedling decreased with increasing UV-A intensity. However, in our results, UV-A intensity did not affect SLW, and this difference seems to be species-specific. Fr treatments had lower SLW than other treatments, such as R, B, R5B5, R3B7, and UV-A. Associated with this, some studies reported that a high R/Fr ratio indicates thin leaves [32,33]. The seedlings under Fr treatments had a larger leaf area, seemingly affecting the low SLW in Fr treatments.

The SPAD value, indicating the chlorophyll content, was the highest in Dini and lowest in Fr treatments (Figure 4D). Yeoung [34] reported that diniconazole applied to Chinese cabbage increased the SPAD value. Son and Oh [35] and Son [36] reported that combined R and B light increases chlorophyll content. Our results showed that combined R and B treatments had higher SPAD values than FL, monochromatic R, and B treatment. It is known to that combined R and B light promotes the plant growth and photosynthesis than monochromatic R and B light [37], because it is the synergistic effect on the absorption of red light in phytochrome and the absorption of blue light in cryptochrome [38]. UV-A treatments had higher SPAD values than FL, monochromatic R and B, and FR treatments, but there was not a significant difference in UV-A intensity. To summarize, combined R and B treatments and UV-A treatments increased leaf area, SLW, and SPAD value, and decreased leaf shape index. Fr treatments increased leaf area and leaf shape index and decreased SLW and SPAD values.

## 4. Conclusions

Growth control of seedlings for inhibiting overgrowth seedlings is important to maintain seedling quality. In this study, we used various light quality for growth control of “Joeunbaekdadagi” cucumber seedlings and to replace the chemical growth regulator. Various light qualities caused many different morphological changes in the cucumber seedlings. The combined R and B treatments and UV-A treatments produced compact plants and increased seedling quality compared with the Dini treatment. Fr treatment increased stem elongation and decreased seedling quality. Especially, R5B5 treatment was effective in decreasing plant height and hypocotyl length and increasing seedling qualities such as compactness, dry matter, and SLW. Thus, using this light quality in CPPS can produce compact plants with better seedling quality and could possibly be used as an alternative to chemical plant growth regulator.

## Figures and Tables

**Table 1:** The composition of the nutrient solution used in the experiment.

Chemical	Concentration (mg·L^−1^)	Chemical	Concentration (mg·L^−1^)
Ca(NO_3_)_2_·4H_2_O	531.00	Fe-EDTA	6.29
KNO_3_	656.50	H_3_BO_3_	0.16
KH_2_PO_4_	170.00	CuSO_4_·5H_2_O	0.02
MgSO_4_·7H_2_O	184.50	MnSO_4_·5H_2_O	0.22
NH_4_NO_3_	8.00	H_2_MoO_4_·2H_2_O	0.01
K_2_SO_4_	3.50	ZnSO_4_·7H_2_O	1.45

**Table 2:** The light intensity of each wavelength in different light quality combinations.

Treatment	PPFD (μmol·m^−2^·s^−1^)				
	300–399 nm	400–499 nm	500–599 nm	600–699 nm	700–799 nm
FL	5.4	70.2	82.2	42.1	3.1
R100	0.4	0.2	0.2	199.2	0.7
B100	0.4	198.5	1.1	0.0	0.0
R5B5	0.4	100.0	0.8	98.8	0.1
R3B7	0.6	138.0	0.9	60.3	0.1
R5B5 + UV 0.2	0.4	100.0	0.8	98.8	0.1
R5B5 + UV 0.4	0.4	100.0	0.8	98.8	0.1
R5B5 + UV 0.6	0.4	100.0	0.8	98.8	0.1
R3B7 + UV 0.2	0.6	138.0	0.9	60.3	0.1
R3B7 + UV 0.4	0.6	138.0	0.9	60.3	0.1
R3B7 + UV 0.6	0.6	138.0	0.9	60.3	0.1
R5B5Fr1	0.4	69.5	0.8	64.9	66.1
R5B5Fr2	0.4	84.9	0.6	82.4	41.1
R5B5Fr3	0.4	82.2	0.6	83.7	27.9
R3B7Fr1	0.4	105.5	0.6	46.6	47.6
R3B7Fr2	0.4	120.6	0.7	52.4	26.1
R3B7Fr3	0.4	124.0	0.7	49.8	24.9

Note: PPFD, photosynthetic photon flux density.

**Table 3:** The effects of the light quality on fresh and dry weights of leaf and stem of “Joeunbaekdadagi” cucumber (*Cucumis sativus* L.) measured at 22 days after treatment.

Treatment	Fresh Weight (g/plant)			Dry Weight (g/plant)		
	Leaf	Stem	Total	Leaf	Stem	Total
FL	1.44 gh ^z^	2.49 g	3.93 f	0.058 g	0.109 g	0.167 j
Dini	1.02 h	1.45 h	2.47 g	0.075 fg	0.184 f	0.259 i
R	1.44 gh	3.37 ef	4.81 f	0.052 g	0.272 e	0.324 hi
B	3.10 f	3.22 fg	6.32 e	0.121 e	0.274 e	0.394 gh
R5B5	2.20 g	5.87 a	8.07 d	0.109 ef	0.506 a	0.616 bcd
R3B7	2.04 g	4.30 d	6.34 e	0.112 ef	0.370 d	0.482 fg
R5B5 + UV 0.2	2.12 g	5.28 abc	7.40 de	0.099 ef	0.479 ab	0.578 cde
R5B5 + UV 0.4	2.01 g	5.48 ab	7.49 de	0.083 efg	0.461 abc	0.545 def
R5B5 + UV 0.6	1.96 g	5.05 a-d	7.01 de	0.086 efg	0.426 bcd	0.513 ef
R3B7 + UV 0.2	1.99 g	4.82 bcd	6.84 de	0.086 efg	0.401 bcd	0.486 efg
R3B7 + UV 0.4	1.89 g	5.14 a-d	7.04 de	0.081 fg	0.397 cd	0.478 fg
R3B7 + UV 0.6	1.95 g	4.89 bcd	6.80 de	0.084 efg	0.393 cd	0.477 fg
R5B5Fr1	9.91 a	4.67 bcd	14.57 a	0.287 bc	0.369 d	0.656 bc
R5B5Fr2	8.99 b	4.69 bcd	13.68 ab	0.279 cd	0.367 d	0.646 bc
R5B5Fr3	6.67 e	4.67 bcd	11.54 c	0.299 bc	0.391 cd	0.691 ab
R3B7Fr1	8.46 bc	4.18 de	12.64 bc	0.320 ab	0.342 de	0.662 bc
R3B7Fr2	7.47 d	4.44 cd	11.91 c	0.249 d	0.370 d	0.619 bcd
R3B7Fr3	8.06 cd	4.90 bcd	12.96 bc	0.347 a	0.422 bcd	0.769 a
Significance	***	***	***	***	***	***

^z^ Mean separation within columns by Duncan’s multiple range test at *p* ≤ 0.05. *** Significant at *p* ≤ 0.001.
